# Capacitive micromachined ultrasound transducers for intravascular ultrasound imaging

**DOI:** 10.1038/s41378-020-0181-z

**Published:** 2020-08-24

**Authors:** Jiaqi Wang, Zhou Zheng, Jasmine Chan, John T. W. Yeow

**Affiliations:** Department of Systems Design Engineering, Faculty of Engineering, University of Waterloo, Waterloo, ON N2L 3G1 Canada

**Keywords:** Electrical and electronic engineering, Engineering

## Abstract

Intravascular ultrasound (IVUS) is a burgeoning imaging technology that provides vital information for the diagnosis of coronary arterial diseases. A significant constituent that enables the IVUS system to attain high-resolution images is the ultrasound transducer, which acts as both a transmitter that sends acoustic waves and a detector that receives the returning signals. Being the most mature form of ultrasound transducer available in the market, piezoelectric transducers have dominated the field of biomedical imaging. However, there are some drawbacks associated with using the traditional piezoelectric ultrasound transducers such as difficulties in the fabrication of high-density arrays, which would aid in the acceleration of the imaging speed and alleviate motion artifact. The advent of microelectromechanical system (MEMS) technology has brought about the development of micromachined ultrasound transducers that would help to address this issue. Apart from the advantage of being able to be fabricated into arrays with lesser complications, the image quality of IVUS can be further enhanced with the easy integration of micromachined ultrasound transducers with complementary metal-oxide-semiconductor (CMOS). This would aid in the mitigation of parasitic capacitance, thereby improving the signal-to-noise. Currently, there are two commonly investigated micromachined ultrasound transducers, piezoelectric micromachined ultrasound transducers (PMUTs) and capacitive micromachined ultrasound transducers (CMUTs). Currently, PMUTs face a significant challenge where the fabricated PMUTs do not function as per their design. Thus, CMUTs with different array configurations have been developed for IVUS. In this paper, the different ultrasound transducers, including conventional-piezoelectric transducers, PMUTs and CMUTs, are reviewed, and a summary of the recent progress of CMUTs for IVUS is presented.

## Introduction

Coronary artery disease (CAD), also called coronary or atherosclerotic heart disease, carries a high mortality rate worldwide. CAD arises from the accumulation of cholesterol and plaques on the arterial inner walls. These plaques slow down the flow of blood to the heart muscle by clogging the artery or by causing vascular functional abnormality^1^. The golden standard to diagnose this disease is invasive coronary angiography that can determine the morphology of the blood vessels to visualize stenosis^2^. However, the view of this method is too limited to detect complex atherosclerotic situations because some lesions are commonly not reflected in the luminal silhouette before they obstruct lumen, which makes it challenging for the assessment of CAD progression and prevention^3^. To include the diagnosis more accurately, optical coherence tomography (OCT), photoacoustic endoscopic imaging and intravascular ultrasound (IVUS) have emerged. OCT is a noninvasive imaging technique that makes use of backscattered light, typically in the infrared range, to acquire live images at near-microscopic resolution (1–2 mm)^4^. During the operating process of OCT, apart from a standard contrast flush to clear the blood column, an extra flush in combination with saline is sometimes adopted, but this may cause damage to kidney^5^. By contrast, photoacoustic endoscopic relies on laser-induced ultrasound to provide images^6,7^. This method breaks the shackles of pure optical imaging that relies on ballistic photons, allowing the sharp contrast of optical images as well as the high-quality imaging at depths of about 2 mm^8–10^. However, due to the inadequate imaging resolution at larger depths, the photoacoustic probe needs to be positioned near the targeted region^11^. Contrary to other techniques, IVUS is able to reach the penetration depth of 4–8 mm inside the blood vessel wall^12^. For this system, the ultrasound probe emits sound waves that echo off the blood vessel walls and return to the system. Real-time signals with different intensities are addressed and analyzed by the console to display a series of longitudinal tomographic images^13–15^. As a cardiovascular (CV) interventional approach, IVUS can build live and high-quality images inside the vessel with minimal side effects^16^. In particular, IVUS is exceptionally competitive in examining arterial wall architecture and lesion morphology^17^. In this review, we mainly discuss the limitations and enhancement of IVUS, thereby enabling IVUS to provide more accurate information for physicians.

Owing to the crucial role of offering imaging information, different ultrasound transducers on the IVUS system will be presented. Currently, conventional-piezoelectric-based transducers are the most widely chosen because of their mature technology, but they specify an inherently small bandwidth^18^. Besides, rigid piezoceramic materials match a higher acoustic impedance when compared to human tissues^18^. Hence, an acoustic impedance matching layer akin to human tissues is added, but the ideal thickness is usually tough to realize^19^. Furthermore, to fabricate high-frequency arrays, transducer elements should be packed tightly together. As a primary approach to fabricate conventional-piezoelectric transducers, mechanical dicing restricts the pitch dimensions and causes the aliasing phenomenon^20^. Since catheters are frequently used in minimally invasive surgery (MIS), the size of the catheter is also a critical factor in reaching the proximity of the imaging objects. Presently, commercially available IVUS catheters are smaller than 6F (ca. 2.0 mm). With the advent of microelectromechanical systems (MEMS) technology^21–24^, micromachined ultrasound transducers can be constructed with a smaller size. Therefore, such transducers can be promising as medical interventional imaging tools. Piezoelectric micromachined ultrasound transducers (PMUTs) share the fabrication strength of integrated circuits but still exhibit narrow bandwidth^25^. When the center frequency increases, the fabricated membrane of PMUTs becomes relatively thin and fragile^26^. This makes the fabrication process of PMUTs more challenging. The development of MEMS technology also stimulates that of capacitive micromachined ultrasound transducers (CMUTs) in many areas, such as medical and therapeutic imaging^27^ and non-destructive testing^28^. Specifically, CMUTs demonstrate numerous advantages in IVUS systems. Compared with conventional-piezoelectric transducers, CMUTs do not need an acoustic impedance matching layer. Instead of mechanical dicing, photolithography technology is adopted to fabricate CMUTs^29^. This technology is well suited for fabricating smaller elements and manipulating high-density CMUT arrays^30^. More importantly, the similarity of the fabrication technology of complementary metal-on-semiconductor (CMOS) and CMUTs allows mutual integration to form CMUT-on-CMOS structure, which reduces parasitic capacitance and improves the signal-to-noise (SNR)^20^.

In this paper, different ultrasound transducers in IVUS will be presented, and the development of IVUS with CMUTs will be discussed in detail. The paper is structured as follows: the next chapter briefly introduces the IVUS catheter. Chapter 3 describes three different types of an ultrasound transducer that are conventional-piezoelectric transducers, PMUTs, and CMUTs, followed by the recent progress of IVUS with CMUTs. Finally, the review concludes by giving an outlook on IVUS with CMUTs relying on the abovementioned sections.

## Intravascular ultrasound catheter

The IVUS catheter is mounted with a miniature ultrasound transducer with a center frequency of 20–40 MHz^31^. Since the choice of frequency associates with the resolution and penetration depth, there is a trade-off between these properties depending on different design requirements^32^. In the practical situation, mechanical and solid-state catheters are frequently used. In the mechanical system, a motor outside the body is used for rotating the drive cable to control the transducer^33^. The drive cable should be as flexible as possible, so to adjust for the curving vessel and accurately position the transducer. For a rotary transducer, the aperture size should also be smaller than the diameter of the catheter. Due to the mechanical binding and the inconsistent rotation of the transducers, nonuniform rotational distortion (NURD) is inevitable and observed for both in vivo body tissues and ex vivo investigations^34^. Another disadvantage is that shadow effects result in the blurring of images on account of the side guidewire. Solid-state catheters address some of these issues. In the solid-state catheter system, a wide range of small transducer arrays are mounted at the catheter tip around the cylinder, and a cross-sectional scan can be conducted by the ultrasonic beam. With the guidewire arranged in the center of the lumen, shadow artifacts can be avoided. However, when transducers are close to tissues, the overlapping transmitted pulses from tissues can produce adverse artifacts called “ring-down artifacts”. The artifacts appear as bright halos of variable thickness and can obscure the image^35^. According to different requirements, mechanical and solid-state catheters can be selectively produced. Currently, the solid-state catheter called “Eagle Eye Platinum ST” has been rolled out by Phillip Volcano, with the center frequency of 20 MHz.

In the following section, ultrasound transducers, the core component to provide imaging information, will be introduced in detail.

## Ultrasound transducers in intravascular ultrasound

Ultrasound transducers play a crucial role in the performance of the IVUS system as they can produce high-frequency sound waves and are able to offer real-time high-resolution Doppler imaging^36^. In this chapter, conventional-piezoelectric transducers, PMUTs and CMUTs in medical ultrasound imaging are presented.

### Conventional-piezoelectric transducer

**Fig. 1:**
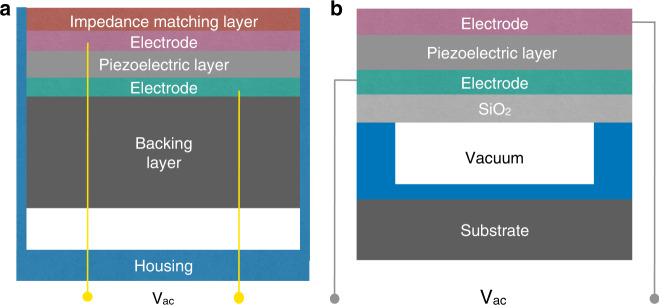
Overview of the cross-sectional schematic of piezoelectric transducers. **a** Conventional-piezoelectric transducer. **b** Piezoelectric micromachined ultrasound transducer. Schematic is not drawn to scale.

The conventional-piezoelectric transducer (Fig. 1a) is an electroacoustic transducer that converts mechanical energy into electrical energy, and vice versa^37^. As an active part, the piezoelectric layer is sandwiched between two electrodes. Two main layers are added to the piezoelectric layer. One is known as the acoustical impedance matching layer that is located on top of the transducer. To achieve better energy transmission between the piezoelectric layer and the medium, the acoustic impedance of piezoelectric materials should be near that of the biological tissues for imaging^38^. The other one is the thick backing layer that can provide mechanical support to the active element and prevent reverberation^39^. To date, depending on the design requirements, the traditional piezoelectric transducer can be commonly produced as single element or arrays.

Due to the mature fabrication technologies, conventional-piezoelectric-based transducers presently dominate the market of IVUS catheters. As can be seen from Table 1, single-element-based ultrasound transducers can be fabricated with the center frequency up to 150 MHz^31^, but only show the penetration depth of <7 mm. Within the focal zone, the transducers can yield a relatively high resolution. Once beyond the zone, the resolution of single-element transducers decreases, thus limiting the frame rate. As a result, it remains challenging for IVUS to obtain real-time and high-quality imaging with a broad field of view. Additionally, such transducers require mechanical scanning, thereby creating a blurred image. For miniaturized phased arrays like one-dimensional (1-D) arrays, they can provide forward-looking (FL) and volumetric ultrasound images with mechanical scanning and combining obtained 2-D planes into 3-D imaging results^40^. However, motion effects can have an impact on this complicated procedure. By contrast, ultrasound transducers based on 2-D multielement arrays can provide volumetric imaging without moving the probe and have demonstrated the lateral resolution of 320 μm at 6 mm penetration depth^41^. However, as the signal acquisition system is tough to connect with wide ranges of elements, the integration of multielement transducers in intravascular catheters remains challenging. Multi-ring arrays have also been explored for IVUS applications^42^, but the limited space in the catheter makes it tough to accommodate these large wire assemblies. Besides, the existing technology to fabricate piezoelectric arrays relies on complicated steps such as high-precision dicing. To solve the above problems, the material type of the piezoelectric transducer is being investigated by global researchers. In particular, the fabrication technology of lead zirconate titanate (PZT) is most mature, and PZT has a high piezoelectric coupling coefficient. Both lead indium niobate-lead magnesium niobate (PMN-PT) and lead indium niobate-lead magnesium niobate-lead titanate (PIN-PMN-PT) can be fabricated with wide bandwidth, but have low curie temperature, resulting in unstable thermal properties during fabrication and operation in IVUS imaging devices^43^. Moreover, most traditional materials are lead-based, which may cause an environmental hazard and may be harmful to human health. Although some piezoelectric materials without lead have been confirmed, the inferior acoustic and electrical features of these novel materials make it difficult to surpass conventional lead-based materials^44,45^. Due to the development of MEMS, piezoelectric-materials-based micromachined ultrasound transducers have attracted much attention.

**Table 1:** Potential ultrasound transducers for IVUS system.

Transducer	Array distributions	Frequency (MHz)	Aperture size	Penetration depth (mm)	Axial resolution	Lateral resolution	Ref.
Conventional-piezoelectric transducer	Single element	60.2	0.45 mm∗0.45 mm	3.1–6.9	24.8 μm	156.1 μm	^89^
	Single element	41	N/A	N/A	43.0 μm	N/A	^43^
	Single element	33.6	0.5 mm∗0.5 mm	5	46.0 μm	231.5 μm	^31^
		91.2		2	21.5 μm	123.5 μm	
		120.0		1	25.7 μm	105.3 μm	
		149.7		0.5	17.2 μm	87.3 μm	
	Phased array	42.6	1 mm∗1 mm	11	118 μm	467 μm	^40^
	2-D array	14	Outer diameter 1.5 mm	6	N/A	320 μm	^41^
	2-D array	5.6	2.5 mm∗6.6 mm	N/A	N/A	N/A	^42^
PMUT	Ring array	6	Side length 600–700 μm	N/A	N/A	N/A	^90^
	2-D array	5	1.1 mm∗6.3 mm	300	500 μm	1 μm	^52^
	2-D array	5	1.5 mm∗1.5 mm	25–35	N/A	N/A	^53^
CMUT	1-D array	35.6	0.3 mm∗1.0 mm	2.4	N/A	277 μm	^66^
	1-D array	9.2	1.73 mm∗1.27 mm	N/A	N/A	N/A	^91^
	1-D array	5	2.392 mm∗ 0.312 mm	10–30	N/A	N/A	^69^
	1-D array	5	Diameter 2.97 mm	71	440 μm	0.12 rad	^68^
		20.8		16	55 μm	0.035 rad	
	2-D dual-ring array	20.1	Outer diameter 1.4 mm	4–8.2	92 μm	251 μm	^74^
	2-D dual-ring array	12	Outer diameter 2.1 mm	4.5	N/A	N/A	^92^

### Piezoelectric micromachined ultrasound transducer

PMUTs have solved some of the issues that traditional transducers cannot address—specifically, the integration with application-specific integrated circuits (ASICs). However, the fabrication of PMUTs remains a challenge because the center frequency is sensitive to the residual stress of the active layer. With the increase of frequency, the fabricated membrane tends to be as thin as possible, which may result in membrane cracking during the fabrication process. Additionally, practical PMUTs usually performs worse than their theoretical design. Figure 1b shows the cross-sectional structure of PMUTs. A PMUT is based on a piezoelectric membrane from a series of patterned layer stack that is deposited on a silicon wafer. The membrane is driven by applying an excitation voltage between the top and bottom electrodes. Transverse stress in the active piezoelectric layer is formed from the applied electric field, which causes the membrane to displace out-of-plane, generating a pressure wave in the outer medium^46^. Different piezoelectric materials have been produced in the PMUTs, such as PZT^47^, zinc oxide (ZnO)^48^, and aluminum nitride (AlN)^49^. With a low operating frequency of kHz range, ZnO-based PMUTs are not suitable for IVUS probes that usually operate in MHz range^50^.

**Fig. 2:**
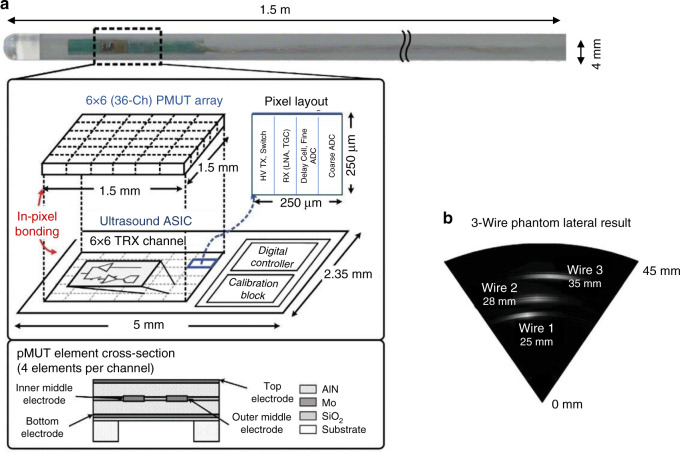
Overview of the PMUT-based ICE system and its phantom results. **a** PMUT-based ICE framework. The 1.5 m ICE tube has a 4 mm diameter. This catheter integrates a 2-D 6 × 6 5MHz 250 μm pitch PMUT array and a 6 × 6 pitch-matched ASIC. **b** In this B-mode picture, the phantom is developed with polydimethylsiloxane (PDMS), where three wires are embedded inside. The phantom is set over the PMUT array, and three wires are in 25, 28, and 35 mm above from the base. Reproduced from ref. ^53^, Copyright © 2019, with the permission of IEEE.

PMUTs show some advantages in the catheterization, such as a small form factor and no need for the polarization voltage to meet the sensitivity requirement. Besides, PMUTs can achieve CMOS integration and have high capacitance with low resistance and poor sensitivity to parasitic capacitance^51^. Presently, PMUT-based catheters are mainly fabricated in the form of intracardiac echocardiography (ICE). As there are some structural similarities between ICE and IVUS catheters, the design of ICE catheters potentially guides that of IVUS catheters. Dausch et al.^52^ fabricated two ICE catheters using 2-D PZT-based PMUT arrays based on 512 elements and 256 elements, respectively. The ICE system was applied for B-mode images where in vivo tests were operated. In the 60° × 60° volume sector, the frame rates of 26 and 31 volumes per second were obtained with scan depths of 10 cm and 8 cm, respectively. This was the first report that presented in vivo live 3-D imaging results based on PMUT arrays. In 2019, Lee et al.^53^ presented an AlN-based PMUT ASIC for portable ICE applications (Fig. 2). They achieved pitch-matched integration of PMUTs on the ASIC. Such integration can help reduce parasitic capacitance for ultrasound transducers in IVUS systems. Low power consumption of 5.37 mW per channel was realized to prevent tissue damage from overheating. Furthermore, the team also presented the imaging of the wire phantom as a potential model in the ICE system. However, there are relatively few studies devoted to the PMUTs in the IVUS system. A possible reason is that the PMUTs in the IVUS system put forward a higher requirement for fabricating the thin membranes. Furthermore, PMUTs have significantly benefited from the development of materials, especially the AlN, that exhibits the low coupling coefficient. Nonetheless, even when enhanced with doping, PMUTs may not be able to satisfy the transmit sensitivity and bandwidth requirements for IVUS^54^. By contrast, high-frequency CMUTs with a large bandwidth may be more suitable for the IVUS system.

### Capacitive micromachined ultrasound transducer

#### Basic structure

**Fig. 3:**
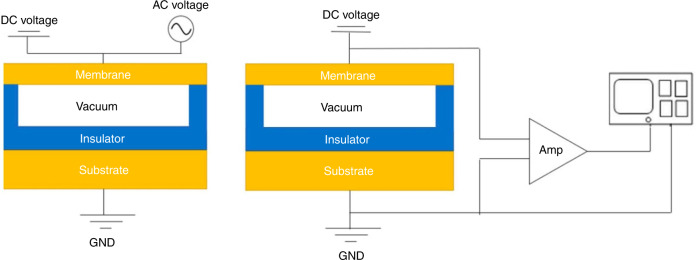
Overview of CMUT structure and different modes. **a** Transmission mode. **b** Receiving mode. Schematic is not drawn to the scale. Reproduced from ref. ^58^, Copyright © 2019, with the permission of Sensors.

CMUTs are MEMS-based devices that can receive and transmit acoustic signals^55^. As seen in Fig. 3, one CMUT cell comprises a thin membrane suspended on top of a vacuum gap^56^. A thin metal layer is formed over the membrane as the top electrode, and the silicon substrate acts as the bottom electrode^57^. An insulation layer is deposited on the silicon substrate to stop mutual contact between two electrodes^58^. Coating materials like low-temperature silicon dioxide (LTO) and low-temperature co-fired ceramic (LTCC) are sometimes used to cover the top electrodes to achieve electrical separation^59^. A CMUT has two working modes. In the transmission mode, both a direct current (DC) voltage and an alternating current (AC) voltage are exerted onto the electrodes^60^. The function of DC bias is to make two electrodes closer, and that of AC makes the membrane vibrate to generate an ultrasonic signal^61^. In the receive mode, a DC voltage is employed across the two electrodes. The gap height modulates with the incident acoustic waves. Consequently, the capacitance of the device changes and the output current is produced. After this, the output current is converted into a voltage signal that can be amplified and detected.

#### CMUT and CMOS integration technologies

The fabrication of CMUTs relies on the fabrication processes of the ubiquitous integrated circuit (IC), thus resulting in highly cost-effective manufacturing. For the CMUT-in-CMOS technology, CMUT and front-end electronics are fabricated in parallel on the same chip and under the same CMOS process^62^. Although this technology is labour- and time-saving, the type and properties of specific material and the layer thickness of the CMUTs are subjected to the restrictions of the CMOS process. More importantly, standardized MEMS techniques allow CMUTs to be integrated on ICs, producing tiny sizes. Therefore, for a tiny transducer area, two CMUT-CMOS integration techniques are commonly adopted. Firstly, the flip-chip Integration process allows the CMUTs and the electric circuits to be optimized separately. During this process, the high-temperature CMUT fabrication processes can be performed independently from CMOS fabrication. Additionally, the stacked CMUT and CMOS circuits allow for a small form factor. However, according to the requirements for flip-chip bonding, the CMUT design must consider through-wafer vias. This means that both the top and bottom electrodes can be accessed by the bonding pads at the bottom of the wafer^63^. As a result, this method is complex. By contrast, the CMUT-on-CMOS process is a more cost-efficient and useful method. For this process, CMUT is fabricated on top of the CMOS electronic circuits, resulting in a small in-plane area and minimal parasitic capacitance caused by electronic traces and interfaces. The main challenge of this technique is that the high fabrication temperature of CMUTs may cause damage to the electronics. However, due to the comprehensive research in low-temperature fabrication processes^64^, CMUT-on-CMOS remains a desirable technology, especially in IVUS systems.

#### Array distributions

In consideration of the convenience for physicians, IVUS imaging catheters are designed with side-looking (SL) and FL functions. SL characteristic is useful in determining the relative position of the blood vessels, and FL is helpful to offer volumetric real-time images in specific vessels that are nearly blocked^65^. To achieve above functions, several configurations of CMUT arrays are constructed, mainly phased arrays, ring arrays, and cylindrical arrays.

#### Phased CMUT array

**Fig. 4:**
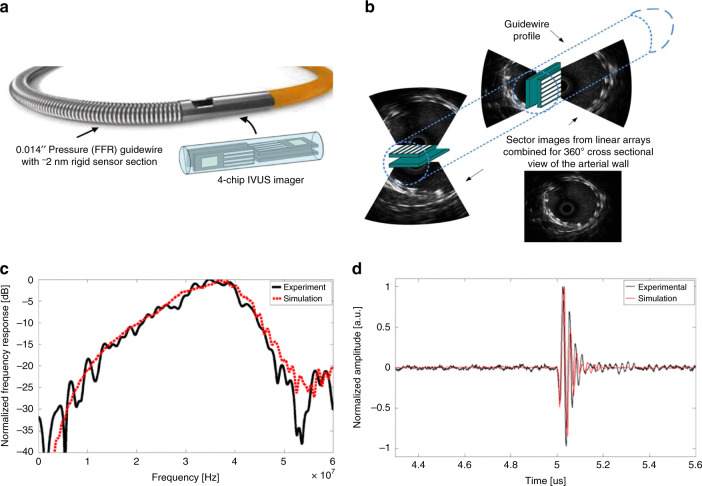
Overview of the side-looking phased CMUT array. **a** The schematic of guidewire-based IVUS catheter. Schematic is not drawn to the scale. **b** The formation process of one cross-section image based on 1-D CMUT arrays forms. **c** The experimental and simulated frequency response with transmission and receiving modes. **d** The experimental and simulated A-scan transient response results. Reproduced from ref. ^66^, Copyright © 2013, with the permission of IEEE.

Phased CMUT arrays contain multiple individual elements that can be controlled separately in a programmable pattern. They can achieve tiny dimensions and high imaging resolution at a low cost. Therefore, some research groups focus on IVUS catheter design with CMUT-phased arrays. For example, Xu et al.^66^ designed and fabricated a 12-element 1-D CMUT-phased array (Fig. 4a). They adopted four 90 degrees 1-D CMUT-phased arrays to form the cross-sectional side-looking imaging of the artery (Fig. 4b). The hydrophone measurements were also carried out to verify the center frequency of 35.6 MHz as well as −3 dB bandwidth at about 10 MHz (Fig. 4c, d). Additionally, with a 300 μm wide aperture size, this design achieved a lateral resolution of 277 μm. This result demonstrates the feasibility of CMUT miniaturization for IVUS catheters. In 2017, Lim et al.^67^ use the same configuration of 12-element CMUT arrays to present a readout interface system-on-chip using only two wires for the guidewire. This reduced-wire operation helped to maintain the mechanics of the guidewires.

**Fig. 5:**
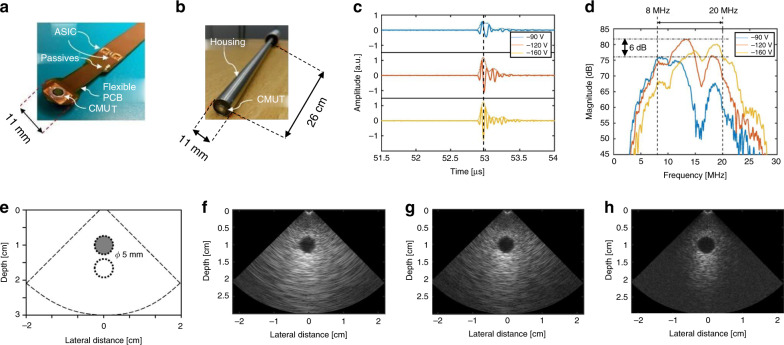
An example of mode-adjustable forward-looking phased CMUT arrays. **a** The picture of CMUT array arranged on the flexible PCB. **b** The CMUT array integrated into a catheter. **c** It displays the pulse-echo response of the CMUT array at −90, −120, and −160 V. **d** Frequency responses of the CMUT array at −90, −120, and −160 V. **e** Schematic of the chronic inflammatory response syndrome phantom imaging result. Schematic is not drawn to the scale. Phantom imaging results in **f** penetration mode, **g** generic mode, and **h** in imaging mode. Reproduced from ref. ^68^, Copyright © 2018, with the permission of Ultrasonics.

The phased array can also satisfy the design requirements of forward-looking ICE and IVUS systems to provide overall and detailed imaging near the catheter probe. A catheter with these considerations was realized by Pekar et al.^68^ based on a 32-element frequency-tunable CMUT-phased array (Fig. 5b). The 32 CMUT elements were split and connected to two 16-channel ASICs using a bendable printed circuit board (PCB) (Fig. 5a). Figure 5c, d showed three time-domain wave pictures from a specific imaging element in the array center. It can be seen in the corresponding spectrums that frequency response moved toward higher frequency with increasing bias voltage. This innovative probe could offer a versatile choice of penetration and resolution mode within one device and could adjust frequencies during a continuous range (Fig. 5e–h). Under the penetration mode, imaging penetration can reach up to 71 mm with 0.44 mm axial resolution and 0.12 rad lateral resolution in tissue-mimicking phantoms. Under the resolution mode, imaging depth is minimum at 16 mm with 0.055 mm axial resolution and 0.035 rad lateral resolution.

Presently, commercial IVUS or ICE catheters still retain numerous interconnections because each channel element needs an individual connection with an external ultrasound system. Nonetheless, the heating of conducting wires damage electronics, and therefore the number of interconnections should be decreased. Jung et al.^69^ reduced the number of wires from 64 to 22. Besides, they fabricated a low-cost CMUT-on-CMOS system on a single chip for the ICE catheter and applied this 9 F (ca. 0.3 cm) catheter to B-mode imaging of wire phantom.

#### Ring CMUT array

1-D phased CMUT arrays can only form cross-sectional imaging of the vessels in the IVUS systems and needed additional steering or pullback tools to achieve 3-D functionality. Contrary to those phased arrays, 2-D single-ring CMUT arrays can simplify the system design. FL CMUT ring arrays can show a wide field of view and mitigate reconstruction artifacts^70^. Additionally, this geometry design optimizes and utilizes the front space of the catheter. In 2004, Demirci et al.^71^ fabricated a 64-element FL single-circular CMUT array. This array demonstrated a 13.5 MHz resonance frequency in air and 24 kPa/V output pressure at the surface of the transducer. Other data of the immersion pulse-echo displayed when operated from 5 to 26 MHz range, and the device presented a 135% fractional bandwidth. This implies ring CMUT arrays were able to provide the characteristic of high-level dynamic range and broad frequency bandwidth. This design also arranged a guidewire to offer Doppler information for detecting blood flow in the intravascular ultrasound systems. To improve the performance of single-ring CMUT arrays, the concept of multiple-ring CMUT arrays was proposed^72^. This dual-ring design could separate transmit and receive mode and improved the quality of harmonic imaging. In this design system, the synthetic phased array processing was also achieved, which means that the multiplexing in multiple firings of array channels was necessary. However, the number of firings should be reduced to realize a high frame rate and mitigate the motion artifacts^73^.

**Fig. 6:**
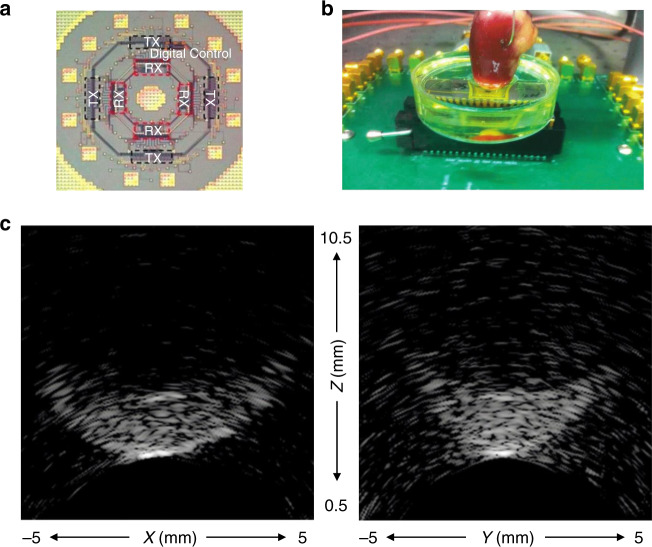
An example of a ring CMUT array, experiment instruments, and imaging results. **a** The schematic of IC for the dual-ring CMUT array. This IC includes receive IC, transmit IC, and the digital control circuitry. Schematic is not drawn to the scale. **b** Experimental device of the CMUT for imaging ex vivo chicken heart phantom. **c** Imaging results of the chicken heart phantom in XZ and YZ planes with 10 mm dimension size. Reproduced from ref. ^74^, Copyright © 2014, with the permission of IEEE.

Ring CMUT arrays can also be achieved on a single chip. Gurun et al.^74^ designed and fabricated a 20 MHz dual-ring monolithic integrated CMUT-on-CMOS system on a single chip (Fig. 6a). A single chip can be only 1 mm, with ten cables. It can simplify the complexity of interconnections and reduces the steps of manufacturing this probe. More importantly, CMUT arrays were directly placed above the CMOS wafer, which reduced the fabrication sophistication and decreased the parasitic capacitance^75^. The arrays included 56 transmit elements in the outer ring and 48 receive elements in the inner ring. Matched axial resolution and the lateral resolution was 92 µm and 251 µm. As shown in Fig. 6b, they also carried out phantom imaging on the chicken heart and collected volumetric imaging data at 60 frames per second. These images showed that 14 dB SNR was obtained after nearly 3–4 mm propagation in different mediums at 20 MHz (Fig. 6c). The result confirms the feasibility of a 3-D real-time volumetric imaging system using CMUT-on-CMOS chip, but there is still a large room for improvement in the imaging SNR and the imaging speed.

#### Cylinder CMUT array

**Fig. 7:**
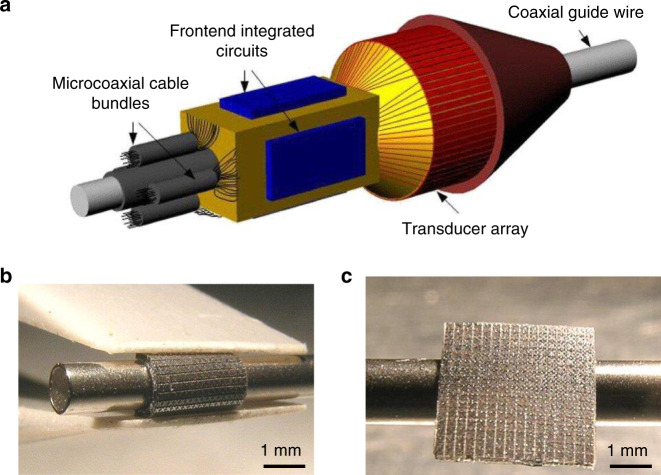
An example of a potential cylindrical CMUT model. **a** It shows the schematic of a side-viewing IVUS imaging probe, including coaxial guidewire, transducer array, front-end electronics, and micro coaxial cable bundles. **b**, **c** are the flexible silicon-based substrate using PDMS. Schematic is not drawn to the scale. Reproduced from ref. ^76^, Copyright © 2008, with the permission of IEEE.

Cylindrical CMUT arrays or conformal external arrays act as a side-viewing model. The fabrication of cylindrical CMUT arrays starts with flexible substrates. These substrates can be curved and to cover the cylinder. Next, they can wrap around the tube tip with ~450 μm radius^76^. Zhuang et al.^76^ displayed a CMUT with the flexible substrate (Fig. 7c) based on refilled PDMS channels (Fig. 7a, b). In the air, the characterized center frequency ranged from 4.3 to 5.0 MHz. Potentially, radial CMUT arrays can combine with FL arrays for enhancing the IVUS system to simplify the diagnostic and surgical procedures.

Researchers also focus on improving the fabrication process and materials of flexible CMUT arrays. For instance, Abgrall et al.^77^ developed a novel photolithography process using lamination technology at low temperature and pressure to enhance the bonding technique. A refined roll-lamination approach was later adopted to produce a transparent flexible CMUT^78^. In 2019, Hah^79^ updated the previous simulation result and displayed a new all-polymer CMUT simulation design for IVUS. The author figured that the current problems of flexible CMUT came from the high-resonant frequency and draw-in voltage^80^. At present, cylindrical CMUT arrays have been tested in the capsule ultrasound (CUS) device^81^. This capsule-shape design guides applying cylindrical CMUT arrays into the IVUS system.

## Discussion

In this paper, ultrasound catheters and transducers of the IVUS system are discussed. Although conventional-piezoelectric transducers are being widely produced, they mainly rely on lead-based materials that are harmful to the environment and humans. By contrast, PMUTs benefit from the development of MEMS technology, but they are also challenging on account of their challenging fabrication process and low working frequency. In particular, CMUTs exhibited unique advantages over other ultrasound transducers, such as wide bandwidth and capability of manipulating high-density arrays. Such features make them promising for IVUS imaging applications.

During the last decade, tremendous progress has been achieved in developing CMUTs and integrating them into IVUS systems. CMUT devices adopt common semiconductor materials that are commonly lead-free. This is advantageous over other lead-based devices. Another effort is the progress of different fabrication processes. For the sacrificial release process, the addition of through-wafer via from the backside allows the fabrication of 2-D arrays with electrical connectivity^82,83^, further enhancing the electrical integration. During wafer-bonding processes, allowing for the charging issue due to its impact on device reliability, some efforts have been made, such as the use of new approaches to form electrodes^84^, the introduction of high-K (dielectric constant) insulators and the improvement of atomic layer deposition^85^. The fabrication technologies of CMUTs provides immense opportunities for the medical market because both the silicon micromachining and wafer-bonding help reduce the expected manufacturing costs. In parallel to fabrication processes, device structures have also been developed like piston and post CMUTs, to enhance average displacement and higher acoustic pressures in transmitting^86,87^. CMUTs and CMOS technologies help chip-size integration, accordingly allowing miniaturization for more sturdy designs. The simultaneous attention to product development and technological improvements is required for better compatibility between CMUTs and the IVUS system. Presently, the frequently used center frequency of CMUTs is commonly 10 MHz. Such frequency is challenging to obtain a high-resolution image. Thus, one pivotal consideration is to fabricate high-frequency arrays. Another underexplored domain is to fabricate a flexible CMUT array, which can offer a large field of view for imaging applications and potentially improves the SNR. Furthermore, this also can help accelerate the imaging speed, alleviate motion artifacts, and reduce the current issue of long diagnostic duration. Additionally, how to form 3-D imaging based on 2-D arrays is a hot topic. Due to the shadow effect caused by mechanical scanning, the fabricated 2-D arrays should avoid a mechanical operation, which is beneficial to the live 3-D imaging.

Considering the multifunction of a future portable system, CMUTs for IVUS imaging should not be a stand-alone application. The CMUT has successfully achieved the measurement of sound speed, flow rate, viscosity, and acoustic impedance in the fluid environment, which provides a prototype as a portable IVUS assistant tool to measure blood density^88^. Additionally, CMUTs can help enhance dual-mode for IVUS associated with other features such as high-intensity focused ultrasound (HIFU) or with OCT imaging. These combinations would enable researchers to explore and diagnose coronary diseases within one process directly. The golden era of portable IVUS system with CMUTs to enhance the treatment and diagnosis of coronary arterial diseases has just begun.

## Supplementary information

Abbreviation words and their full name

Editorial Summary
