# Supplementary material for: Capacitive micromachined ultrasound transducers for intravascular ultrasound imaging
Source: Microsyst Nanoeng. 2020 Aug 24;6:73. doi: 10.1038/s41378-020-0181-z (PMC8433336; doi:10.1038/s41378-020-0181-z)
Supplement: Supplementary file 1 — Abbreviation words and their full name [file 41378_2020_181_MOESM1_ESM.docx]

**Table 1** Abbreviation words and their full name

| Abbreviation | Full name |
| --- | --- |
| IVUS | Intravascular ultrasound |
| MEMS | Microelectromechanical system |
| CMOS | Complete metal-oxide-semiconductor |
| PMUT | Piezoelectric micromachined ultrasound transducer |
| CMUT | Capacitive micromachined ultrasound transducer |
| CAD | Coronary artery disease |
| 2-D | Two-dimensional |
| 3-D | Three-dimensional |
| OCT | Optical coherence tomography |
| CV | Cardiovascular |
| VH | Virtual histology |
| MIS | Minimally invasive surgery |
| SNR | Signal-to-noise |
| NURD | Non-uniform rotational distortion |
| FL | Forward-looking |
| 1-D | One-dimensional |
| PZT | Lead zirconate titanate |
| PMN-PT | Lead indium niobate-lead magnesium niobate |
| PIN-PMN-PT | Lead indium niobate-lead magnesium niobate-lead titanate |
| ASIC | Application-specific circuit |
| ZnO | Zinc oxide |
| AlN | Aluminum nitride |
| ICE | Intracardiac echocardiography |
| PDMS | Polydimethylsiloxane |
| LTO | Low-temperature silicon dioxide |
| LTCC | Low-temperature co-fired ceramic |
| DC | Direct current |
| AC | Alternating current |
| NEP | Noise equivalent pressure |
| IC | Integrated circuit |
| SL | Side-looking |
| FFR | Fractional flow reserve |
| PCB | Printed circuit board |
| CUS | Capsule ultrasound |
| HIFU | High-intensity focused ultrasound |
