# Supplementary material for: Capacitive micromachined ultrasound transducers for intravascular ultrasound imaging
Source: Microsyst Nanoeng. 2020 Aug 24;6:73. doi: 10.1038/s41378-020-0181-z (PMC8433336; doi:10.1038/s41378-020-0181-z)
Supplement: Supplementary file 2 — Editorial Summary [file 41378_2020_181_MOESM2_ESM.docx]

# *Microsystems & Nanoengineering*

Imaging: Micro-machined ultrasound transducers for intravascular ultrasound imaging

Ultrasound transducers are employed in intravascular ultrasound (IVUS; imaging technology used in diagnosing coronary arterial disease), and of the types of transducers currently available, capacitive micro-machined ultrasound transducers (CMUTs) show the greatest promise. In IVUS systems, ultrasound transducers, which transmit acoustic waves and detect the returning signals, are important for obtaining high-resolution images. A team headed by John T.W. Yeow at the University of Waterloo, Canada undertook a review of conventional piezoelectric (allowing energy conversion from the mechanical to electrical domain) transducers, piezoelectric micro-machined ultrasound transducers (PMUTs), and CMUTs. Currently, PMUTs face significant challenges regarding their fabrication process and low working frequency. By contrast, the authors identify considerable merits with CMUTs. The authors believe that the golden era of portable IVUS systems with CMUTs for diagnosing and treating coronary arterial disease has just begun.

Related article manuscript number: MICRONANO-01169

Article title: Capacitive Micromachined Ultrasound Transducers for Intravascular Ultrasound Imaging

Corresponding author and affiliation/s: John T.W. Yeow, University of Waterloo, Systems design engineering, Waterloo, Ontario, Canada

**About your Editorial Summary — please read**

**Before approving this Editorial Summary, please carefully check that (1) the summary text lists the correct author(s) and (2) the spelling and order of all author names and affiliations are correct.**

This **Editorial Summary** is based on your manuscript that was recently accepted for publication in *Microsystems & Nanoengineering*. It provides a non-specialist audience with a synopsis of your key research outcomes and conclusions. This value-added service provided by Springer Nature is designed to raise interest in your research across the broader community.

Springer Nature will publish the summary on the journal’s website, and it will be freely available under a under the CC BY licence (Creative Commons Attribution v4.0 International Licence) (see the journal website for details). We encourage you to re-use the summary to bring attention to your research; for example, you can host it on your own website and share it via social-networking platforms. Please attribute the summary to *Microsystems & Nanoengineering* and your article (e.g. by providing a link to your article) and do not make derivatives.

Please note that to maximise the usefulness of these summaries they must follow several stringent guidelines:
-- Spelling, punctuation and style are set according to *Nature* editorial guidelines. As this summary is aimed at non-expert readers, some concepts and technical terms will be simplified.
-- Total length must be no more than 135 words. It is likely that not all points in the paper will be covered.
-- The first sentence must be no more than 280 characters, including spaces, to allow use on microblogging sites.
-- The headline must consist of a brief generic subject identifier followed by a short description. No more than 10 words in total.

Please contact the editorial office ([mems_nano@mail.ie.ac.cn](mailto:mems_nano@mail.ie.ac.cn)) immediately with corrections should you find any factual errors in this Editorial Summary.
